# Test–retest reliability and follow‐up of muscle magnetic resonance elastography in adults with and without muscle diseases

**DOI:** 10.1002/jcsm.13528

**Published:** 2024-06-24

**Authors:** Bram De Wel, Lotte Huysmans, Ronald Peeters, Stefan Ghysels, Kris Byloos, Guido Putzeys, Frederik Maes, Patrick Dupont, Kristl G. Claeys

**Affiliations:** ^1^ Department of Neurology University Hospitals Leuven Leuven Belgium; ^2^ Department of Neurosciences Laboratory for Muscle Diseases and Neuropathies, KU Leuven, and Leuven Brain Institute (LBI) Leuven Belgium; ^3^ Medical Imaging Research Centre University Hospitals Leuven Leuven Belgium; ^4^ Department ESAT PSI, KU Leuven Leuven Belgium; ^5^ Department of Radiology University Hospitals Leuven Leuven Belgium; ^6^ Department of Neurosciences Laboratory for Cognitive Neurology, KU Leuven, and Leuven Brain Institute (LBI) Leuven Belgium

**Keywords:** Becker muscular dystrophy, MRE, muscle stiffness, quantitative MRI, outcome measure

## Abstract

**Background:**

We investigated the potential of magnetic resonance elastography (MRE) stiffness measurements in skeletal muscles as an outcome measure, by determining its test–retest reliability, as well as its sensitivity to change in a longitudinal follow‐up study.

**Methods:**

We assessed test–retest reliability of muscle MRE in 20 subjects with (*n* = 5) and without (*n* = 15) muscle diseases and compared this to Dixon proton density fat fraction (PDFF) and volume measurements. Next, we measured MRE muscle stiffness in 21 adults with Becker muscular dystrophy (BMD) and 21 age‐matched healthy controls at baseline, and after 9 and 18 months. We compared two different methods of analysing MRE data in this study: ‘Method A’ used the stiffness maps generated by the Philips MRE software, and ‘Method B’ applied a custom‐made procedure based on wavelength measurements on the MRE images.

**Results:**

Intraclass correlation coefficients (ICC) of muscle stiffness ranged from good (0.83 for left vastus medialis, *P* < 0.001) to poor (0.19 for right rectus femoris, *P* = 0.212) for the examined thigh muscles with Method A, but we did not find a significant test–retest reliability with Method B (*P* > 0.050 for all). The ICC of muscle PDFF and volume measurements was excellent (>0.90; *P* < 0.001) for all muscles. At baseline, the average stiffness of all thigh muscles was significantly lower in adults with BMD than in controls for both Method A (−0.2 kPa, *P* = 0.025) and Method B (−0.6 kPa, *P* < 0.001). Regardless of which method was used, there was no significant difference in the evolution of muscle stiffness in patients and controls over 18 months.

**Conclusions:**

Test–retest reliability of muscle MRE using a simple 2D technique was suboptimal, and did not reliably measure muscle stiffness changes in adults with BMD as compared with controls over 18 months. While the results provide motivation for testing more advanced 3D MRE methods, we conclude that the simple 2D MRE implementation used in this study is not suitable as an outcome measure for characterizing thigh muscle in clinical trials.

## Introduction

Dixon magnetic resonance imaging (MRI) of intramuscular proton density fat fraction (PDFF) is widely recognized as a sensitive and objective outcome measure in muscle diseases.^1^, ^2^, ^3^, ^4^, ^5^ However, Dixon imaging only visualizes the amount of intramuscular fat, and not fibrosis, which is a key hallmark of muscular dystrophies and directly impacts muscle function.^6^ Previous research on muscle biopsies in Duchenne muscular dystrophy (DMD) showed that skeletal muscle fibrosis was the most accurate marker for poor clinical outcome in the long term.^7^ Consequently, quantitative imaging of muscle fibrosis could have important repercussions for patients with neuromuscular disorders, both in clinical trials and clinical practice, resulting in a highly unmet need for such a non‐invasive outcome measure at present.^8^

Magnetic resonance elastography (MRE) is a long‐established technique to assess fibrosis of the liver.^9^, ^10^ MRE determines the stiffness of tissues by measuring the displacement caused by a wave propagation of a vibrating source placed over the studied tissue.^10^, ^11^, ^12^ This is an indirect method of evaluating fibrosis, as fibrotic tissues have an increased stiffness compared with healthy tissues. In mouse models, skeletal muscle MRE distinguished between diseased mdx (mouse model for DMD) and healthy mice, and muscle stiffness correlated strongly with collagen fraction on histological examination.^13^, ^14^ MRE has sporadically been applied to skeletal muscles in humans in the past decades, for example, to show that muscle stiffness increases during muscle contraction.^15^, ^16^, ^17^ Some studies also aimed to quantify stiffness in different muscles to create an atlas for future reference, and evaluate differences in stiffness between healthy and diseased muscles.^11^, ^18^, ^19^, ^20^, ^21^, ^22^ However, as data on test–retest reliability of muscle MRE are scarce, there is no consensus on the methodology of acquiring or analysing MRE images, and there is a lack of longitudinal follow‐up data.^11^, ^23^, ^24^, ^25^

In this study, we first investigate the test–retest reliability of muscle MRE imaging and compare this to the reliability of Dixon PDFF and muscle volume measurements. Next, we perform a prospective longitudinal MRE imaging study in adults with Becker muscular dystrophy (BMD) and age‐matched healthy controls, to determine the sensitivity of muscle stiffness changes over time. Both are crucial aspects to determine the potential of muscle MRE as a non‐invasive outcome measure for future application in clinical trials.

## Methods

### Patients and study design

To evaluate test–retest reliability and feasibility of MRE, we recruited both healthy volunteers and patients with muscle diseases (limb‐girdle muscular dystrophy recessive type 12 or type 9 and sporadic inclusion body myositis) to undergo two identical multimodal MRI scans—including T1, Dixon and MRE images of the upper legs—with a 1‐week interval.

To evaluate if MRE sequences could detect a change in thigh muscle stiffness over time, we performed the same multimodal MRI in a different group of subjects at baseline, and after 9 and 18 months: adult patients with BMD and age‐matched healthy male controls. Patients with BMD were symptomatic and ambulatory. Individuals unable to undergo an MRI (e.g., pacemaker) were not eligible for participation. Additionally, all participants had to refrain from straining physical activity in the 7 days preceding all study visits. This was a precautionary measure to ensure optimal reproducibility, as one previous study showed that stiffness could remain increased for up to 7 days after vigorous exercise in some muscles of the lower leg.^26^ Written informed consent was obtained from all participants and the study was approved by the Ethics Committee Research UZ/KU Leuven.

### Magnetic resonance imaging data acquisition

**Figure 1:**
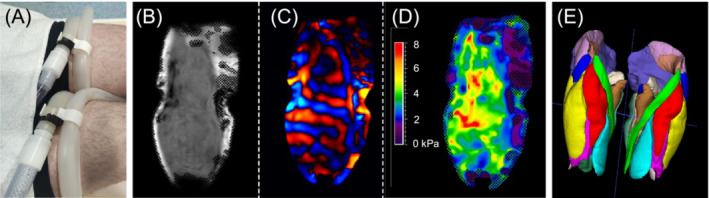
MR elastography set‐up and images. (A) Photograph of the silicone tubes wrapped around a patient's thighs on the MRI table, which are connected to a vibrating source in the adjacent room. (B) Sagittal slice of the native magnitude MRE image over the left vastus lateralis muscle. (C) MRE wave image, which visualizes tissue displacement from propagating vibration waves and is calculated from the native magnitude and phase images. (D) Stiffness map calculated by the Philips MRE software package from the wave image. The colour indicates the stiffness of each voxel in kPa (legend included). The shaded areas indicate that the stiffness was not accurately measurable in that location and were therefore excluded from analysis. (E) The 3D muscle segmentation map of this patient that was used for analysis of individual muscles. The muscle in yellow is the vastus lateralis.

All subjects were scanned in the same 1.5 Tesla Philips Ingenia MRI scanner (Philips Medical Systems, Best, the Netherlands) in a supine, feet‐first position with one posterior and two anterior coils for signal acquisition. Patients were told to relax and the legs were positioned flat and unloaded on the MRI table and immobilized with sandbags, placed laterally against the legs, to ensure that they were completely relaxed. For MRE acquisition, we developed a custom‐made driver for transmission of vibrations onto the legs in analogy with the methods described in Bensamoun et al. (i.e., a silicon tube wrapped around the leg and connected to a loudspeaker).^11^ This driver consisted of a fenestrated rigid silicon tube inside a flexible thin silicon tube, which was wrapped around the thigh at midpoint distance from the anterior superior iliac spine to the upper border of the patella (Figure 1A). This landmark was chosen so that vibrations would be transmitted optimally over the most voluminous part of the thighs, and because these anatomical landmarks are easily measurable in a consistent manner. The silicon tubes were manually tightened with a clasp system to ensure a good contact with the leg. We analysed the following muscles: rectus femoris, vastus lateralis/intermedius/medialis, sartorius, gracilis, adductor longus/magnus, biceps femoris long and short head, semimembranosus, and semitendinosus. Other thigh muscles were not (sufficiently) present in the region where vibration waves were clearly detectable. The rigid inner tube prevented the driver from collapsing under the weight of the subject's leg, and the flexible thin outer tube over the fenestration optimally transmitted vibrations onto the leg. The silicon tubes were connected to a pneumatic vibrating source in a room adjacent to the MRI, which transmitted vibrations at 60 Hertz (Hz). This frequency is used in our centre because it visually delivers optimal shear waves in the thigh muscles with our set‐up and is within the range that is typically used in the literature (25–150 Hz).^25^, ^27^

The MRE acquisition [repetition time (TR) 50 ms, echo time (TE) 20 ms, flip angle (FA) 20°, 16 sagittal slices with 10 mm slice thickness and 1.6 mm interslice gap, field of view (FOV) 450 × 197 × 184 mm^3^, voxel size = 1.5 × 4.0 mm^2^] consisted of a sagittal phase‐contrast MRI sequence of one leg at a time in the sagittal plane with a motion‐encoding gradient oscillating at the same frequency as the driver (i.e., 60 Hz). We also acquired axial T1 turbo spin echo sequences [repetition time (TR) 412 ms, echo time (TE) 4.0 ms, 30 slices with 8 mm slice thickness and 1 mm interslice gap, field of view (FOV) 300 × 455 mm^2^, voxel size = 1.4 × 1.4 mm^2^] and 6‐point Dixon fast imaging in 3D (TR/TE1/delta TE = 9.2/1.36/1.3 ms, flip angle 12°, 125 slices, slice thickness 2 mm, FOV 450 × 394 × 252 mm^3^, matrix 320 × 280 × 125, voxel size 1.2 × 1.2 × 2 mm^3^). These are the standard mDixon_QUANT protocol parameters of our 1.5 T Philips MRI scanner because they produce a good signal‐to‐noise ratio and have a short acquisition time. However, as they can incur T1‐weighting we corrected for this in post‐processing with the formulas described in Liu et al.^28^ We acquired three Dixon imaging stacks from the iliac crest to the tibial plateau, each overlapping by 20 slices (40 mm) to ensure that B0 inhomogeneity artefacts that can occur at the outer edges of the Dixon MRI images could be excluded from the analysis. Total scan time was 45 min.

### Magnetic resonance imaging data analysis

First, we obtained individual 3D segmentations of all thigh muscles from origin to insertion, by using a custom‐made convolutional neural network (CNN) for semi‐automated segmentation of the out‐of‐phase Dixon images. Next, we used a custom‐made MATLAB script (MathWorks, Natick, MA, USA) to generate PDFF images out of the Dixon fat and water images as PDFF (%) = fat image/(fat + water image) and calculate the PDFF and volume of each muscle by using the 3D segmentations. This workflow is described in detail in previous studies.^5^, ^29^, ^30^ Patients with a total thigh PDFF of <10% were considered to have a normal Dixon MRI. This cutoff was defined as the mean total thigh PDFF of healthy controls + 2.5 standard deviations.

Regarding the MRE data, the MRI scanner acquired native magnitude and phase MRE images (Figure 1B). The Philips MRE software package rendered wave images (Figure 1C), and stiffness maps (Figure 1D) in which for each voxel of the MRE image a stiffness value in kPa is determined. We compared two different methods of analysing MRE data in this study:

‘Method A’ calculated the stiffness of individual muscles from the stiffness map generated by the Philips software. We co‐registered the 3D muscle segmentations acquired on the Dixon images with the MRE images, which allowed for the calculation of the average muscle stiffness of each measured thigh muscle, using a custom‐made MATLAB script (Figure 1E). The Philips MRE software automatically discarded voxels in regions where wave propagation was not clearly measurable due to technical issues or wave refractions (shaded areas in Figure 1D). Additionally, we discarded voxels with the 5% lowest and 5% highest stiffness values because outliers were often still present.

In ‘Method B’, we calculated muscle stiffness similar to what is usually done in the literature, with less reliance on an MRI vendor software package.^11^, ^15^, ^18^, ^23^ The shear stiffness is typically calculated with the following formula: $\mu =\rho {\left(\mathrm{f\lambda}\right)}^{2}$, which is the density of muscle tissue ρ (assumed at 1000 kg/m^3^ by convention) times the squared product of the frequency f of the induced vibrations (60 Hz in this study) and the measured wavelength λ.^25^ Consequently, by measuring the wavelength in each muscle on the acquired wave images (Figure 1C), the stiffness is readily calculated. Because wave propagation occurs along the direction of each individual muscle (fibre), this means that the wavelength should be measured in a different direction along the length of each individual thigh muscle, as measurements of oblique waves can artificially elevate the measured stiffness.^23^, ^24^ Therefore, we first calculated the main proximo‐distal axis/direction of each individual muscle by using a 3D segmentation model of the entire muscle. Next, we calculated the slice with maximum overlap between the MRE measurement and the segmentation for each muscle. In this slice, we then measured the wavelength for the investigated muscle along its determined main proximo‐distal axis. The average wavelength over the eight acquired wave phases (describing the wave at 0, 45, 90, 135, 180, 225, 270, and 315 degrees) was then used to calculate muscle stiffness.^21^ However, because skeletal muscle is an anisotropic, non‐linearly viscoelastic tissue with a complex geometry and many borders, the waves propagating through the muscle may not follow a regular sinusoidal waveform. Instead, multiple different wavelengths are often discernible in the complex wave image of each muscle. For optimal reproducibility, we did not measure wavelength manually in each muscle, but applied a Fourier power analysis to calculate which wavelength was most prevalent in the wave image of each muscle. For this power analysis, wave lengths of over 50 mm were excluded as they were deemed likely to be an artefact.

### Statistical analysis

We used RStudio® Desktop (Open‐Source Licence, version 4.1.2) for all statistical analyses. Significance level was determined at α = 0.05. To assess test–retest reliability, we determined intraclass correlation coefficients (ICC) with a single rater, two‐way mixed effects model with absolute agreement. Coefficients of >0.90 are interpreted as an indication of excellent reliability, >0.75 as good, 0.50–0.75 as moderate, and <0.50 as poor reliability.^31^ To further evaluate reliability we reported Pearson and Spearman correlation coefficients depending on normality of the underlying data, as assessed by the Shapiro–Wilk test.^32^ These were also reported for correlations of muscle stiffness with age and PDFF. We used *t*‐tests and Wilcoxon ranked‐sum tests both to compare patient and control groups at baseline and to evaluate the differences in these groups over 9 and 18 months. Holm's method was used to correct for multiple testing.^33^ Finally, we used the standardized response mean (SRM) to assess the effect size of muscle stiffness changes over 9 and 18 months.

## Results

### Patient characteristics

Subject characteristics for both the test–retest reliability part of the study, and the longitudinal follow‐up part of patients with BMD, are detailed in Table 1. In short, to measure test–retest reliability we included 20 adults, of which five had different muscle diseases, and for the longitudinal follow‐up study, we included 21 adult patients with BMD and 21 age‐ and sex‐matched healthy controls. None of the participants reported discomfort from the vibrations during the MRE acquisition, and only one subject mentioned mild paresthesia in the legs, which disappeared immediately after he got up from the MRI table. All subjects were still ambulatory, but seven (33.3%) patients with BMD required walking aids. Sixteen patients (76.2%) with BMD carried a deletion, three patients (14.2%) a point mutation, one patient (4.8%) a duplication, and one patient (4.8%) an intronic splice variant in the *DMD* gene. Nine (42.9%) of the patients' genetic defects included exon 45, eight (38.1%) were downstream of exon 45 and four (19%) were upstream. No patients were treated with corticosteroids. Three patients with BMD did not complete the follow‐up study visit at month 9 (one due to a hip fracture with surgery, and two due to personal reasons) and were therefore not included in the longitudinal analysis. The patient with the hip fracture completed the visit at month 18, but MRE data were not reliable due to artefacts from the femoral nail.

**Table 1:** Baseline subject characteristics

	Test–retest study		Longitudinal BMD study	
Demographics	Patients (*n* = 5)	Controls (*n* = 15)	Patients (*n* = 21)	Controls (*n* = 21)
Gender				
Male	4 (80%)	5 (33.3%)	21 (100%)	21(100%)
Female	1 (20%)	10 (66.7%)	0	0
Age (years)	53 [40–69]	45 [26–58]	40 [18–66]	40 [18–66]
Age at symptom onset (years)	39 [28–57]	‐	13 [4–50]	‐
Disease				
BMD	0	‐	21 (100%)	‐
LGMDR12	3 (60%)	‐	‐	‐
LGMDR9	1 (20%)	‐	‐	‐
sIBM	1 (20%)	‐	‐	‐
Current BMI (kg/m^2^)	29.5 ± 2.5	25.4 ± 5.5	25.8 ± 6.0	25.1 ± 2.9
Baseline total thigh PDFF %	36.3 ± 23.1	6.7 ± 3.3	35.3 ± 25.8	5.4 ± 1.7

Data are shown as n (%), median [range], or as mean ± standard deviation. Total thigh PDFF at baseline differed significantly (*P* < 0.001) between the matched BMD patient and control groups, but age and BMI were not significantly different.

BMD, Becker muscular dystrophy; BMI, body mass index; LGMDR12/9, limb‐girdle muscular dystrophy recessive type 12 or type 9; PDFF, proton density fat fraction; sIBM, sporadic inclusion body myositis.

### Test–retest reliability of magnetic resonance elastography, proton density fat fraction, and volume measurements

**Figure 2:**
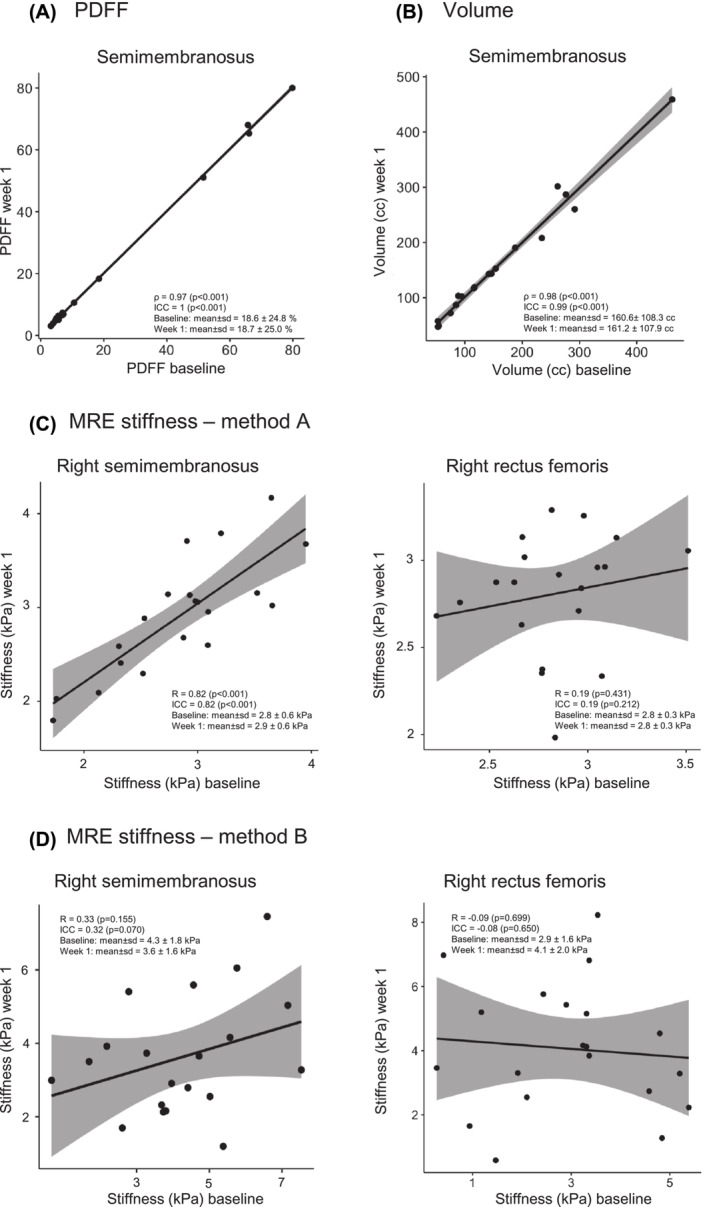
Test–retest reliability of PDFF, volume and MRE measurements in the semimembranosus muscle. Reliability is best when all measurements at baseline and week 1 are equal and fall on the line of identity and the confidence interval (grey area) is small. (A) Correlation between baseline and week 1 PDFF measurements in the semimembranosus muscles. (B) Correlation between baseline and week 1 volume measurements in the semimembranosus muscles. (C) Correlation between baseline and week 1 MRE stiffness measurements with Method A (Philips MRE software package) in the semimembranosus and rectus femoris muscles of the right leg as an example of a muscle with a good and a poor test–retest reliability, respectively. (D) Correlation between baseline and week 1 MRE stiffness measurements with Method B (custom MATLAB script with measurements of vibration wavelength in individual muscles) in the semimembranosus and rectus femoris muscles of the right leg.

We evaluated the test–retest reliability of MRE muscle stiffness measurements in both legs separately with two different methods of analysis, for which the ICC and Pearson/Spearman correlation coefficients are detailed in Table 2. With Method A, we calculated an important variation in test–retest reliability, ranging from good and significant in some muscles (e.g., semimembranosus) to poor and insignificant in others (e.g., rectus femoris; Figure 2). With Method B, there was no significant correlation between stiffness measured at baseline and 1 week later for all muscles.

**Table 2:** Test–retest reliability of MRE muscle stiffness analyses

	Method A				Method B			
Muscles	ICC right	R/ρ right	ICC left	R/ρ left	ICC right	R/ρ right	ICC left	R/ρ left
Rectus femoris	0.19	0.19	0.51	0.51	−0.08	−0.09	−0.15	−0.22
Vastus lateralis	**0.82**	**0.82**	**0.65**	**0.66**	0.24	0.16	−0.01	−0.01
Vastus intermedius	0.38	0.37	0.40	**0.41**	0.21	0.20	0.21	0.20
Vastus medialis	**0.67**	**0.66**	**0.83**	**0.83**	0.36	0.31	−0.41	−0.42
Sartorius	0.28	0.28	0.43	0.45	0.20	0.19	0.27	0.27
Adductor longus	**0.62**	**0.68**	0.33	0.36	−0.05	0.11	0.43	0.54
Adductor magnus	**0.77**	**0.78**	0.51	0.56	0.09	0.15	−0.01	0.01
Gracilis	0.20	0.26	**0.57**	**0.59**	0.40	0.48	0.22	0.23
Semimembranosus	**0.82**	**0.82**	**0.78**	**0.73**	0.32	0.33	−0.22	−0.29
Semitendinosus	**0.66**	**0.66**	**0.63**	**0.62**	0.14	0.05	−0.51	−0.48
Biceps femoris caput longus	**0.72**	**0.72**	**0.58**	0.57	0.17	0.06	−0.02	−0.02
Biceps femoris caput brevis	**0.62**	**0.65**	**0.63**	**0.68**	0.32	0.33	−0.06	−0.19

Method A analysed muscle stiffness with the Philips MRE software package and Method B with a custom method of wavelength measurement detailed in the methods section. A two‐way mixed effect model with absolute agreement was used to calculate the ICC. Pearson and Spearman correlation coefficients are reported in the R/ρ columns. Significant correlation coefficients (after Holm's correction) are shown in bold.

ICC, intraclass correlation coefficient; R/ρ, Pearson or Spearman correlation coefficients.

We observed excellent test–retest reliability of both PDFF and volume measurements on Dixon MRI of the thigh muscles in healthy volunteers and in patients, as indicated by the ICC and Pearson/Spearman correlations (Table 3, Figure 2). Baseline PDFF was significantly higher in patients than in controls (*P* < 0.001, Table 1), and baseline total thigh muscle volume was lower in patients (2,467 ± 1,147 cc) than in controls (3,903 ± 2,268 cc), although this was not significant (*P* = 0.098).

**Table 3:** Test–retest reliability of PDFF and muscle volume analyses

	PDFF		Muscle volume	
Muscles	ICC	Correlation coefficient	ICC	Correlation coefficient
Rectus femoris	1.00	0.91	1.00	1.00
Vastus lateralis	1.00	0.89	1.00	1.00
Vastus intermedius	1.00	0.89	1.00	0.99
Vastus medialis	1.00	0.98	1.00	0.99
Sartorius	1.00	1.00	1.00	1.00
Pectineus	1.00	0.85	0.99	0.99
Adductor brevis	1.00	0.92	0.99	0.99
Adductor longus	1.00	0.97	1.00	0.99
Adductor magnus	1.00	0.98	1.00	0.97
Gracilis	1.00	0.99	1.00	0.99
Semimembranosus	1.00	0.97	0.99	0.98
Semitendinosus	1.00	0.97	1.00	0.99
Biceps femoris caput longus	1.00	0.97	1.00	0.99
Biceps femoris caput brevis	1.00	0.96	0.98	0.99
Tensor fascia lata	1.00	0.98	0.99	0.99
Gluteus maximus	1.00	0.99	0.99	0.99
Gluteus medius	0.98	0.83	0.95	0.90
Gluteus minimus	1.00	0.89	0.97	0.94

A two‐way mixed effect model with absolute agreement was used to calculate the ICC. Pearson or Spearman correlation coefficients are reported depending on normality of the data. *P*‐values were always <0.001 and remained significant after Holm's correction.

ICC, intraclass correlation coefficient; PDFF, proton density fat fraction.

### Magnetic resonance elastography measurements in Becker muscular dystrophy patients and controls at baseline and months 9 and 18

**Figure 3:**
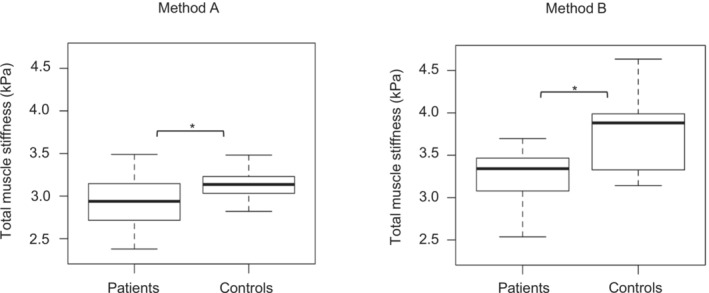
Differences in baseline muscle stiffness in adults with BMD and controls. Baseline, muscle stiffness was significantly lower in the BMD patient group than in the matched control group when comparing a non‐weighted average of all measured thigh muscles with both Method A (−0.2 kPa, *P* = 0.025) and Method B (−0.6 kPa, *P* < 0.001).

At baseline, muscle stiffness was significantly lower in the BMD patient group than in the matched control group (Figure 3) when comparing a non‐weighted average of all measured thigh muscles with both Method A (−0.2 kPa, *P* = 0.025) and Method B (−0.6 kPa, *P* < 0.001). With Method A, we found a significant increase in muscle stiffness of the biceps femoris long head muscle in patients, and of the rectus femoris and adductor magnus muscles in controls after 18 months (Table 4), but there was no significant difference between the increases in the patient and control groups for these muscles. Data for the change in muscle stiffness over 9 months are shown in Supplementary Table S1. With Method B, we did not detect any significant changes in muscle stiffness in patients or controls over 9‐ and 18‐months follow‐up. For patients, sensitivity to change over 18 months was low to moderate with Method A, reflected by a SRM of 0.01–0.79, and low in Method B with a SRM of 0.07–0.48 for all muscles.

**Table 4:** Change in thigh muscle stiffness in patients with BMD and matched controls over 18 months

Stiffness kPa	Method A			Method B		
	Baseline stiffness	Month 18	*P*‐value	Baseline stiffness	Month 18	*P*‐value
Patients						
Rectus femoris	2.8 ± 0.4	+0.1 ± 0.4	0.151	3.0 ± 1.2	−0.3 ± 1.6	0.485
Vastus lateralis	3.1 ± 0.4	+0.1 ± 0.3	0.119	3.7 ± 1.6	+0.9 ± 1.8	0.057
Vastus intermedius	3.1 ± 0.3	0.0 ± 0.3	0.969	4.5 ± 1.6	−0.4 ± 2.0	0.386
Vastus medialis	2.6 ± 0.4	+0.2 ± 0.3	0.043	2.2 ± 1.2	+0.2 ± 1.7	0.644
Sartorius	3.2 ± 0.3	+0.1 ± 0.3	0.091	3.5 ± 1.4	−0.7 ± 1.6	0.094
Adductor longus	2.8 ± 0.6	0.0 ± 0.5	0.650	3.0 ± 1.0	−0.1 ± 1.3	0.779
Adductor magnus	2.3 ± 0.5	+0.1 ± 0.3	0.481	2.8 ± 1.4	−0.2 ± 2.0	0.724
Gracilis	3.6 ± 0.8	0.0 ± 0.4	0.369	3.6 ± 1.5	+0.3 ± 1.7	0.511
Semimembranosus	2.4 ± 0.6	+0.1 ± 0.4	0.568	3.4 ± 1.2	+0.5 ± 2.3	0.359
Semitendinosus	3.4 ± 0.8	+0.2 ± 0.3	0.006	3.0 ± 1.9	+0.6 ± 2.4	0.262
Biceps femoris long head	2.7 ± 0.4	+0.4 ± 0.5	**0.004**	3.4 ± 1.7	−−0.5 ± 2.9	0.482
Biceps femoris short head	3.1 ± 0.5	0.0 ± 0.5	0.936	2.7 ± 1.5	−0.4 ± 1.9	0.449
Controls						
Rectus femoris	2.9 ± 0.2	+0.2 ± 0.2	**0.001**	3.5 ± 1.4	+0.3 ± 1.8	0.488
Vastus lateralis	3.5 ± 0.3	+0.1 ± 0.2	0.016	4.3 ± 1.9	−0.9 ± 2.0	0.157
Vastus intermedius	3.1 ± 0.3	+0.1 ± 0.3	0.185	4.9 ± 1.5	−0.3 ± 1.9	0.524
Vastus medialis	2.8 ± 0.2	+0.2 ± 0.2	0.004	3.8 ± 1.8	−0.5 ± 2.1	0.279
Sartorius	3.4 ± 0.4	+0.2 ± 0.4	0.098	3.3 ± 1.5	+0.5 ± 2.0	0.286
Adductor longus	3.0 ± 0.3	+0.1 ± 0.3	0.173	4.1 ± 1.2	+0.5 ± 2.0	0.305
Adductor magnus	2.5 ± 0.2	+0.2 ± 0.2	**<0.001**	2.9 ± 1.5	+0.7 ± 2.5	0.256
Gracilis	4.3 ± 0.5	+0.1 ± 0.7	0.579	4.5 ± 1.5	+0.6 ± 1.8	0.135
Semimembranosus	2.7 ± 0.4	0.0 ± 0.4	0.708	3.7 ± 1.3	+0.2 ± 1.9	0.594
Semitendinosus	3.6 ± 0.4	+0.1 ± 0.2	0.011	3.8 ± 2.0	−0.6 ± 2.5	0.274
Biceps femoris long head	3.0 ± 0.4	+0.1 ± 0.2	0.058	3.6 ± 1.5	−0.1 ± 2.1	0.759
Biceps femoris short head	3.1 ± 0.5	+0.1 ± 0.4	0.335	3.3 ± 2.0	−1.0 ± 2.5	0.085

Data at baseline are shown as mean ± SD and at month 18 as mean ± SD of the change from baseline. *P*‐values for the difference between baseline and month 18 are shown. *P*‐values in bold are significant after Holm's correction. For the difference between month 9 and baseline, none of the *P*‐values were significant (except for the adductor magnus in controls with Method A: *P* = 0.002).

BMD, Becker muscular dystrophy; SD, standard deviation.

When patients with normal PDFF MRI scans (and thus no visible disease on MRI; *n* = 5) were excluded from the analysis, the results remained unchanged with both methods.

### Correlation of muscle stiffness to age and proton density fat fraction in patients with Becker muscular dystrophy

With Method A, there was a significant inverse correlation at baseline between age and the non‐weighted average stiffness of all measured thigh muscles in both patients with BMD (*r* = −0.50, *P* = 0.036) and healthy controls (*r* = −0.54, *P* = 0.012), indicating a decrease in muscle stiffness with increasing age. With Method B, there was a similar correlation between thigh muscle stiffness and age for healthy controls (*r* = −0.56, *P* = 0.009), but this was not significant for patients with BMD.

There was a significant, moderately strong, inverse correlation between PDFF and muscle stiffness as measured with Method A in all muscles at baseline in patients, ranging from ρ = −0.53 to ρ = −0.83 (*P* < 0.01 for all), except in the rectus femoris and biceps femoris muscles. Additionally, there was a moderate positive correlation between age and total thigh PDFF (ρ = 0.51, *P* = 0.018). This indicates that muscle stiffness decreased when PDFF and age increased in patients with BMD. With Method B, no correlation was found between muscle stiffness and PDFF in patients with BMD. Regardless of which method was used to measure muscle stiffness, there was also no correlation with PDFF in any muscle in the control group.

## Discussion

We evaluated test–retest reliability for two different methods of measuring muscle stiffness with MRE imaging and showed that it varied from good to poor between muscles, in contrast to a consistently excellent reproducibility of PDFF and muscle volume measurements with Dixon MRI. We also observed that average thigh muscle stiffness was lower in patients with BMD than in matched healthy controls, but we could not detect a significant difference between the change in muscle stiffness in patients and controls over 18 months of follow‐up. Muscle stiffness decreased with increasing age and higher muscle fat replacement (PDFF) in patients with BMD.

### Reliability of magnetic resonance elastography muscle stiffness measurements was suboptimal

To our knowledge, this was the first study with the objective to evaluate test–retest reliability of muscle MRE. There is only one study that reported having rescanned two healthy individuals and described important variability in one of them.^34^

Thigh muscle stiffness measurements with Method A, which calculated stiffness with the Philips MRE software package, showed a test–retest reliability that was good for some muscles, but only moderate or poor for others. It is important to note that the Philips MRE software package was designed for quantification of stiffness in liver tissue, which has very different mechanical properties than muscle tissue. Similarly, MRI vendor software packages to calculate PDFF from Dixon images were also not developed for—or validated in—muscle tissue, which has led to warnings that it should not be assumed that these packages give acceptable results outside their intended terms of use.^4^ The lack of transparency concerning the exact underlying algorithm of these software packages poses another problem.

This explains why in most MRE studies in the literature, muscle stiffness is calculated by manually measuring the wavelength in each muscle (Method B). However, this methodology has its own issues.

First, it has been shown that depending on where in the muscle the wavelength is measured, more centrally of peripherally in the muscle, stiffness can vary by more than 30%.^23^ To some extent this reflects the true differences in tension and stiffness present in different regions of the muscle, and is therefore not necessarily an indication of a methodological error. For this reason, we did not measure wavelengths manually, but automated the process to consistently calculate the major axis of the muscle part with maximum overlap with the MRE measurement in a certain slice as well as the determination of the final wavelength (see Method B).

In addition, as detailed in the methods section, determination of the correct wavelength in a muscle is not as straightforward as might be assumed because waves often do not follow a regular sinusoidal waveform in thigh muscle tissue.^21^ Because we expected inter‐ and intra‐rater variability to be high (and thus reproducibility to be low) for a manual measurement based on a subjective estimation of the principal underlying wavelength in a complex waveform (as is usually done), we opted to automate this process and calculated the most frequently detected wavelength for our stiffness calculations.^23^ However, it is certainly possible that far more advanced algorithms are required for this analysis, which, for example, consider the complex geometry of the muscles of the thigh and filter the wave refractions that occur due to the finite dimensions of muscles. Indeed, despite these precautions, Method B performed worse than the Philips MRE software package in the sense that it did not show a significant test–retest reliability for any of the muscles we evaluated, and it also did not show correlations between stiffness and age or PDFF.

Finally, it is also clear that the formula that is used for stiffness calculations based on the wavelength, $\mu =\rho {\left(\mathrm{f\lambda}\right)}^{2}$, is an oversimplification, as it wrongly assumes that skeletal muscle is a homogeneous, isotropic, linearly elastic, and incompressible tissue. Only limited progress has been made so far in devising more complex analysis methods that for example consider muscle tissue anisotropy (e.g., with diffusion tensor imaging (DTI)), but it is certainly plausible that this could lead to more reproducible results in the future.^35^, ^36^

Regardless of which methodology is used, another possible reason for the inferior reproducibility of MRE in comparison with Dixon PDFF or muscle volume measurements could be that muscle stiffness is a far more dynamic parameter than fat replacement. For example, if the subjects' muscles are slightly contracted (i.e., not completely relaxed) on the MRI table, the measured muscle stiffness may vary.^11^, ^19^, ^37^ Indeed, the concept of pre‐stress in muscles from static or quasi‐static deformations caused by tensile or compressive loading, which is inherent to biological tissues, has been shown to have an important effect on wavelength and should be modelled for in the calculation of muscle stiffness.^17^ Another example is the influence of physical exertion in the hours to days preceding the MRI scan on leg muscle stiffness reported in the literature.^26^, ^38^

### Interpretation of muscle stiffness as a biomarker

Both MRE methods in this study found a significantly lower muscle stiffness in patients with BMD than in controls.^21^ This might seem paradoxical (in mouse models a higher muscle collagen fraction (fibrosis) correlated with increased muscle stiffness) but may be explained by a relatively more important muscle fat replacement than presence of fibrosis in adults with BMD.^13^ Indeed, as was previously reported, we also found that muscle stiffness decreased significantly when intramuscular PDFF increased, because fat tissue has a lower stiffness than muscle tissue.^24^, ^39^

This implies that we can expect muscle stiffness to either increase or decrease, depending on the underlying disease process. For example, muscle stiffness was shown to be increased in patients with spastic paraplegia.^20^ Children with DMD were also shown to have a higher muscle stiffness than healthy controls, possibly due to a more aggressive disease progression (with more fibrosis) than in BMD, and the fact that the study population was younger.^22^, ^39^ Interestingly, these children had a lower muscle stiffness than controls during muscle contraction, indicating disrupted contractile properties in DMD.

The interpretation of MRE muscle stiffness is thus complicated because it is not only determined by muscle fibrosis, but by all the pathophysiological changes in muscle tissue, such as muscle fat replacement, but most likely also oedema or inflammation. Therefore, while MRE muscle stiffness can be an interesting marker, its role in measuring fibrosis in muscle tissue is limited compared with more homogeneous tissues such as the liver, due to the varying interplay of different histopathological processes.

### Limitations

It is difficult to compare MRE results between studies in the literature. The few centres that study muscle MRE each use a distinct custom‐made driver set‐up to induce vibrations and many different vibration frequencies are used, which can influence results.^40^ Nevertheless, muscle stiffness values were in the same range as reported in the literature, and Method A revealed similar correlations between stiffness, age, and PDFF as were previously reported.^24^ This suggests that MRE measurements in other centres could also have a similarly suboptimal test–retest reliability, which should certainly be investigated in future studies. A limitation to the test–retest reliability analysis in this study is the small sample size.

We tried to standardize wavelength measurements in Method B to decrease bias from a manual rater. However, this meant that wavelengths were not always measured over the part of the muscle with the most clearly propagating vibration waves. Additionally, because shear waves propagate in three dimensions in the thigh anatomy evaluated in this study, the simple 2D analysis methods that were used do not appear to be adequate to reliably assess muscle stiffness in a clinical setting. Given the poor test–retest reliability of Method B, the results of the longitudinal analysis with this method should be interpreted with care.

Of course, test–retest reliability could also have been influenced by the experimental set‐up. For example, contact pressure between the vibrating tubes and the leg was not quantitatively monitored or kept within a pre‐specified range (the tubes were manually tightened to ensure a snug fit), which could in theory influence stiffness measurements. Additionally, we mentioned that stiffness measured in skeletal muscle is dominated by the state of passive tension/loading, and active contraction. In this study, we measured muscle stiffness in a single unloaded state, but multiple measurements in with small variations in positioning, support, and driver position would have been informative.

Finally, we could not detect a difference in the change of muscle stiffness over time in patients with BMD as compared with controls. However, this finding is influenced by the poor test–retest reliability, and we did not perform a formal sample size or power calculation. It could also be due to the relatively short follow‐up period of 18 months. Indeed, a recent study in patients with BMD showed that the amount of fibrosis on muscle biopsies did not change significantly over 1 year.^41^ A longer follow‐up, or evaluation of a muscle disease with a more rapid progression of fibrosis (e.g., DMD) might be indicated.

### Future directions

The simple methods for quantifying muscle stiffness using (2D) MRE, as applied in this study, were not adequate in a clinical setting. The way forward is to perform a true 3D analysis by using the 3D wavefield and analysing stiffness at each voxel by using an inversion algorithm. From a physical point of view, this would lead to a better estimation of muscle stiffness, but from a clinical point of view, it of course remains to be seen if quantification using such an analysis is reliable, reproducible and robust in patients. This should be evaluated in future studies.

## Conclusions

We conclude that muscle MRE is an interesting and promising technique to measure muscle stiffness, but that the experimental setup and methods of analysis evaluated in this study are not reliable enough to apply this technique as an outcome measure in a clinical trial. Indeed, test–retest reliability was not consistent, and muscle stiffness did not change in patients with BMD as compared with controls over 18 months follow‐up. Future studies should also include test–retest reliability and explore analysis methods that correctly account for the three‐dimensional propagation of shear waves.

## Funding

This study received research funding from the patient organization Association Belge contre les Maladies neuro‐Musculaires (ABMM) and Klinische Onderzoeks‐ en OpleidingsRaad (KOOR) of University Hospitals Leuven and the Kan‐GO! Fund of KU Leuven. BDW is supported by the Fund for Scientific Research Flanders (FWO, PhD fellowship fundamental research grant number 1159121N). The work of FM and LH is supported in part by the Internal Funds KU Leuven under Grant C24/18/047 and by the Flemish Government under the “Onderzoeksprogramma Artificiële Intelligentie (AI) Vlaanderen” programme.

## Conflict of interest

KGC is Chairholder of the Emil von Behring Chair for Neuromuscular and Neurodegenerative Disorders by CSL Behring, member of the European Reference Network for Rare Neuromuscular Diseases (ERN EURO‐NMD) and of the European Reference Network for Rare Neurological Diseases (ERN‐RND). None of the authors report disclosures relevant to the manuscript.

## Supporting information


**Table S1.** Supporting Information.
