# Supplementary material for: Test–retest reliability and follow‐up of muscle magnetic resonance elastography in adults with and without muscle diseases
Source: J Cachexia Sarcopenia Muscle. 2024 Jun 24;15(5):1761–71. doi: 10.1002/jcsm.13528 (PMC11446706; doi:10.1002/jcsm.13528)
Supplement: Supplementary file 1 — Table S1. Supporting Information. [file JCSM-15-1761-s001.docx]

**Table 4.** Change in thigh muscle stiffness in patients with BMD and matched controls over 9 months

| **Stiffness kPa** | **Method A** | | | **Method B** | | |
| --- | --- | --- | --- | --- | --- | --- |
| **Patients** | **Baseline**  **Stiffness** | **Month 9**  **Change in stiffness** | **p-value** | **Baseline**  **Stiffness** | **Month 9**  **Change in stiffness** | **p-value** |
| Rectus femoris | 2.8±0.4 | + 0.2±0.3 | 0.009 | 3.0±1.2 | - 0.2±1.5 | 0.568 |
| Vastus lateralis | 3.1±0.4 | + 0.1±0.3 | 0.161 | 3.7±1.6 | + 0.3±2.0 | 0.524 |
| Vastus intermedius | 3.1±0.3 | 0.0±0.3 | 0.962 | 4.5±1.6 | - 0.6±2.4 | 0.334 |
| Vastus medialis | 2.6±0.4 | + 0.1±0.3 | 0.225 | 2.2±1.2 | + 0.2±2.0 | 0.661 |
| Sartorius | 3.2±0.3 | + 0.1±0.3 | 0.195 | 3.5±1.4 | - 0.5±2.3 | 0.361 |
| Adductor longus | 2.8±0.6 | 0.0±0.4 | 0.999 | 3.0±1.0 | + 0.2±1.4 | 0.533 |
| Adductor magnus | 2.3±0.5 | + 0.1±0.3 | 0.196 | 2.8±1.4 | 0.0±2.1 | 0.952 |
| Gracilis | 3.6±0.8 | + 0.3±0.5 | 0.043 | 3.6±1.5 | 0.0±1.8 | 0.924 |
| Semimembranosus | 2.4±0.6 | 0.0±0.3 | 0.780 | 3.4±1.2 | - 0.1±2.0 | 0.897 |
| Semitendinosus | 3.4±0.8 | 0.0±0.3 | 0.525 | 3.0±1.9 | + 0.3±2.8 | 0.608 |
| Biceps femoris long head | 2.7±0.4 | 0.0±0.4 | 0.941 | 3.4±1.7 | - 0.7±2.5 | 0.223 |
| Biceps femoris short head | 3.1±0.5 | 0.0±0.5 | 0.798 | 2.7±1.5 | - 0.7±1.8 | 0.094 |
| **Controls** |  | | |  | | |
| Rectus femoris | 2.9±0.2 | + 0.2±0.3 | 0.013 | 3.5±1.4 | 0.0±1.9 | 0.683 |
| Vastus lateralis | 3.5±0.3 | 0.0±0.3 | 0.304 | 4.3±1.9 | - 1.1±2.4 | 0.056 |
| Vastus intermedius | 3.1±0.3 | + 0.1±0.3 | 0.395 | 4.9±1.5 | - 0.6±2.4 | 0.239 |
| Vastus medialis | 2.8±0.2 | + 0.1±0.2 | 0.040 | 3.8±1.8 | - 0.5±2.5 | 0.406 |
| Sartorius | 3.4±0.4 | + 0.2±0.4 | 0.076 | 3.3±1.5 | 0.0±1.9 | 0.975 |
| Adductor longus | 3.0±0.3 | + 0.1±0.4 | 0.101 | 4.1±1.2 | - 0.3±1.7 | 0.488 |
| Adductor magnus | 2.5±0.2 | + 0.2±0.2 | **0.002** | 2.9±1.5 | + 0.5±2.5 | 0.339 |
| Gracilis | 4.3±0.5 | + 0.1±0.8 | 0.576 | 4.5±1.5 | - 0.2±2.1 | 0.726 |
| Semimembranosus | 2.7±0.4 | 0.0±0.3 | 0.452 | 3.7±1.3 | + 0.2±1.9 | 0.661 |
| Semitendinosus | 3.6±0.4 | + 0.1±0.3 | 0.362 | 3.8±2.0 | - 1.0±2.8 | 0.103 |
| Biceps femoris long head | 3.0±0.4 | + 0.2±0.3 | 0.007 | 3.6±1.5 | - 0.4±2.1 | 0.400 |
| Biceps femoris short head | 3.1±0.5 | 0.0±0.4 | 0.613 | 3.3±2.0 | - 0.1±3.2 | 0.854 |

Data at baseline are shown as mean±SD standard deviation and at month 9 as mean±SD of the change from baseline. P-value in bold is significant after Holm’s correction. Abbreviations: BMD, Becker Muscular Dystrophy; kPa, kilo pascal; SD, standard deviation.
